# Evidence for Loss in Identity, De-Differentiation, and *Trans*-Differentiation of Islet β-Cells in Type 2 Diabetes

**DOI:** 10.3389/fgene.2017.00035

**Published:** 2017-03-29

**Authors:** Chad S. Hunter, Roland W. Stein

**Affiliations:** ^1^Division of Endocrinology, Diabetes and Metabolism, Comprehensive Diabetes Center and Department of Medicine, University of Alabama at BirminghamBirmingham, AL, USA; ^2^Department of Molecular Physiology and Biophysics, Vanderbilt UniversityNashville, TN, USA

**Keywords:** diabetes mellitus, type 2, β-cell, dedifferentiation, trans-differentiation, epigenetics, transcription factors, islets of Langerhans

## Abstract

The two main types of diabetes mellitus have distinct etiologies, yet a similar outcome: loss of islet β-cell function that is solely responsible for the secretion of the insulin hormone to reduce elevated plasma glucose toward euglycemic levels. Type 1 diabetes (T1D) has traditionally been characterized by autoimmune-mediated β-cell death leading to insulin-dependence, whereas type 2 diabetes (T2D) has hallmarks of peripheral insulin resistance, β-cell dysfunction, and cell death. However, a growing body of evidence suggests that, especially during T2D, key components of β-cell failure involves: (1) loss of cell identity, specifically proteins associated with mature cell function (e.g., insulin and transcription factors like MAFA, PDX1, and NKX6.1), as well as (2) de-differentiation, defined by regression to a progenitor or stem cell-like state. New technologies have allowed the field to compare islet cell characteristics from normal human donors to those under pathophysiological conditions by single cell RNA-Sequencing and through epigenetic analysis. This has revealed a remarkable level of heterogeneity among histologically defined “insulin-positive” β-cells. These results not only suggest that these β-cell subsets have different responses to insulin secretagogues, but that defining their unique gene expression and epigenetic modification profiles will offer opportunities to develop cellular therapeutics to enrich/maintain certain subsets for correcting pathological glucose levels. In this review, we will summarize the recent literature describing how β-cell heterogeneity and plasticity may be influenced in T2D, and various possible avenues of therapeutic intervention.

## Introduction

Over the past three decades, diabetes mellitus has become a worldwide epidemic, with health-care and economic burdens climbing dramatically (Ashcroft and Rorsman, 2012; Guariguata et al., 2014). At the epicenter of diabetes is the β-cell, the most prevalent cell type within the islets of Langerhans, and the only insulin-producing cell in the body, with the actions of this hormone essential for lowering blood glucose levels to maintain euglycemia. T1D results in insulin-dependency after autoimmune mediated β-cell destruction, whereas the more prevalent T2D is a complex metabolic disorder resulting from insulin resistance and progressive β-cell inactivation. Regardless of etiology, both forms of diabetes are underpinned by loss of glucose stimulated insulin secretion and concomitant hyperglycemia. In the setting of T2D progression, prolonged elevation in glucose and/or fatty acid levels leads to a failure of β-cell insulin production, and secretion, potentially followed by reduced cell survival; this condition is termed as gluco-lipotoxicity (reviewed in Poitout et al., 2010).

Although islet β-cell death is a clear outcome in T1D, recent evidence points to more complex mechanisms that affect β-cell identity and function in T2D. A change in identity is defined as reduced expression of proteins or functional changes essential to a cell type, which in this case includes insulin and essential β-cell-enriched transcription factors. This contrasts from de-differentiation, wherein the β-cell is converted into earlier progenitor state, which in rodent models can included loss of insulin expression and gain of Neurogenin 3 (i.e., Ngn3) and Nanog transcription factor production. For example, it was generally believed that the obesity and insulin resistance stress conditions associated with T2D led to islet β-cell failure cell death and reduced insulin^+^ cell mass. However, we will discuss growing evidence suggesting that, as T2D progresses, β-cells do not die, but lose insulin expression, possibly de-differentiating toward a progenitor state, and then into other islet cell types. At a minimum, there is evidence for loss of β-cell identity during the course of the disease (a topic recently reviewed in Brereton et al., 2016). Although less convincing, there is also evidence for altered endocrine identity during T1D. Here, we will assess the latest reports regarding β-cell heterogeneity, which collectively suggest that there are islet insulin^+^ cell populations with varying functionality and signature marker expression levels. We will also discuss how the paradigms of β-cell life and death during T2D have changed in recent years to include greater appreciation for cellular identity and plasticity.

## Islet β-cell identity and heterogeneity

In a classic sense, β-cells have been defined by insulin expression and controlled secretion under high glucose conditions. Indeed, one might refine the definition to also include the requisite expression of key β-cell transcriptional regulators, for example MafA, Nkx6.1, and Pdx1 (Guo et al., 2013; Schaffer et al., 2013; Gao et al., 2014), as well as the cellular machinery responsible for glucose-stimulated insulin secretion (e.g., Glut2, Gck, Kcnj11) (Prentki et al., 2013). However, several lines of evidence now challenge us to further modify this definition. New technologies, including single-cell transcriptomics and mass cytometry, have allowed the field to “drill deeper” into the β-cell mass to more definitively reveal the long-suspected heterogeneity within the insulin^+^ population (Salomon and Meda, 1986; Pipeleers, 1992; Van Schravendijk et al., 1992), especially across species and in non-diabetic vs. T2D donors (Segerstolpe et al., 2016; Wang et al., 2016a,b; Xin et al., 2016). Several recent resource publications shared the single cell transcriptomes of human islet cells under diabetic and non-diabetic conditions (Segerstolpe et al., 2016; Wang et al., 2016b; Xin et al., 2016).

Segerstolpe et al. utilized single-cell RNA-Sequencing profiling to identify five clusters of insulin^+^ cells, two of which had varying levels of *RBP4* and *FFAR4* expression, encoding proteins impacting insulin resistance and release, respectively (Segerstolpe et al., 2016). Further, this study identified genes that were dysregulated in non-diabetic vs. T2D β-cells. *FXYD2* (encoding a Na/K-ATPase subunit) was the most significantly downregulated gene in T2D β-cells, which can influence glucose tolerance and insulin levels in mice (Arystarkhova et al., 2013). Conversely, *GPD2* (involved in mitochondrial metabolism) and *LEPROTL1* (also called endospanin-2, impacts localization of the leptin and GH receptors) were upregulated. Wang et al. compared single-cell transcriptomes across non-diseased, T1D, T2D, and juvenile human islet samples. Interestingly, β-cell gene signatures of adult T2D samples were less defined than in non-diseased adults, with resemblance to less mature juvenile cells (Wang et al., 2016b). These data demonstrate that β-cell gene expression differences exist between healthy and T2D populations. However, it is unclear precisely what the defining functional molecular signatures are, due to the early and limited nature of these studies.

**Figure 1:**
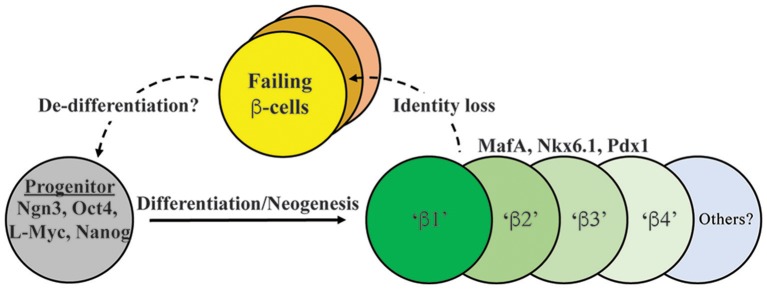
**Model depicting both endocrine progenitor differentiation into a heterogeneous population of β-cells within human islets, and potential pathways of cell inactivation recently described in T2D**. Inactivation likely involves mechanisms that result in loss/reduction β-cell identity marker expression (e.g., MafA, Nkx6.1, Pdx1, insulin) as well induction of cell failure signatures (e.g., ALDH1A3; Kim-Muller et al., 2016). β1–4 cells represent at least some of the functionally distinct β-cell populations in human islets, with our model suggesting that failing T2D islets will be composed of several (e.g., β3 and β4; Dorrell et al., 2016). In addition, there is evidence in (at least) mouse islets that failing β-cells can de-differentiate to progenitor-like cells expressing Ngn3, Nanog, L-Myc, and Oct4 (Talchai et al., 2012).

It is appreciated that islet β-cells have distinct subtype markers within normal and T2D populations. Rat β-cells with increased insulin secretion capacity were found to express higher levels of PSA-NCAM (a cell adhesion molecule) and CDH1/E-Cadherin (Bernard-Kargar et al., 2001; Bosco et al., 2007), whereas human cells express variable levels of *VMAT2* (encoding a monoamine transporter) and *DKK3* (encoding a Wnt signaling modulator) (Hermann et al., 2007; Saisho et al., 2008). More recently, Dorrell et al. utilized cell-surface recognizing antibodies to reveal that human β-cells can be sub-divided into four sub-types (i.e., β1–β4) based upon ST8SIA1 (a ganglioside synthase) and CD9 (a cell surface glycoprotein) levels (Dorrell et al., 2016) (Figure 1). These subtypes had both variable abundance (e.g., β1 > β4) and insulin secretion activity (i.e., β1 > β2–4) under normal conditions. Moreover, the less glucose-responsive β3–β4 cell populations also appeared to become more prevalent in T2D islet samples, presumably representing a state of compromised β-cell activity. In addition, islet β-cell heterogeneity was observed in mouse cell subpopulations due to expression of *Flattop* (*Fltp*), a Wnt/PCP effector gene (Bader et al., 2016). Using a *Fltp* knock-in reporter mouse line (i.e., FVR), Bader et al. found temporal increases in expression, such that 80% of adult Nkx6.1^+^ β-cells were *Fltp*^+^, arising from more proliferative FVR^−^ precursor β-cells. Through cell sorting and comparative mRNA expression and functional profiling, it was proposed that Fltp^+^ (FVR^+^) cells represent a mature β-cell population, with characteristically high insulin levels, enriched mitochondrial activity, elevated metabolic function, and expression of defining cell gene expression signatures. However, *Fltp* knockout mice (i.e., *Fltp*^*ZV*/*ZV*^) had only small, albeit significant defects in fasting blood glucose and plasma insulin levels (Bader et al., 2016), implying that Fltp is a key marker, but not a determinant of mature β-cells.

A newly defined biomarker of “failing” T2D cells appears to involve induction of *aldehyde dehydrogenase 1 isoform A3* (*ALDH1A3*) (Kim-Muller et al., 2016). Thus, upregulated ALDH1A3 levels (normally only produced in progenitors during embryogenesis) were observed in adult islet β-cells from FoxO (1, 3a, 4) triple knockout mice, and also found in the dysfunctional β-cell populations in *db*/*db, Nkx6.1*, and *MafA*-deficient mouse models (Taylor et al., 2013; Hang et al., 2014; Kim-Muller et al., 2016). Sorted ALDH1A3^+^ cells were less glucose responsive than cells lacking ALDH1A3, had reduced levels of mature β-cell markers (e.g., *Glucokinase, MafA*), concomitant increases in progenitor markers (e.g., *Rfx6, Rfx7, Mlxipl*), and mitochondrial dysfunction (Kim-Muller et al., 2016). Although still unclear, some evidence suggests that ALDH1A3 also does not directly cause β-cell dysfunction.

Interestingly, Johnston et al. have revealed another level of islet β-cell heterogeneity upon identifying “hub” β-cells (i.e., ~1–10% of the islet cells) that exert a disproportionate control on the broader β-cell “follower” population (Johnston et al., 2016). The mouse hub cells are intermingled with other islet β-cells but have reduced marks of β-cell maturity, including lower insulin content, reduced Pdx1, and nearly absent Nkx6.1. Using targeted cell laser illumination methods, the hub cells were shown to be essential to glucose-induced Ca^2+^ signaling, and by extension, insulin secretion. (Technical limitations precluded directly analyzing islet insulin secretion levels.) Strikingly, incubating islets in a proinflammatory cytokine cocktail (IL-1β, IL-6, TNFα) impaired hub cell number, providing evidence that the diabetic milieu would compromise islet secretion dynamics. However, the molecular and functional properties of human islet hub cells are still poorly defined as is their relationship to the many recently defined and distinct β-cell populations. Collectively, these studies highlight the heterogeneity of islet β-cells and illustrates how the gluco-lipotoxic conditions associated with diabetes could influence their prevalence by changing in β-cell identity or differentiation states.

## Islet cell plasticity

**Figure 2:**
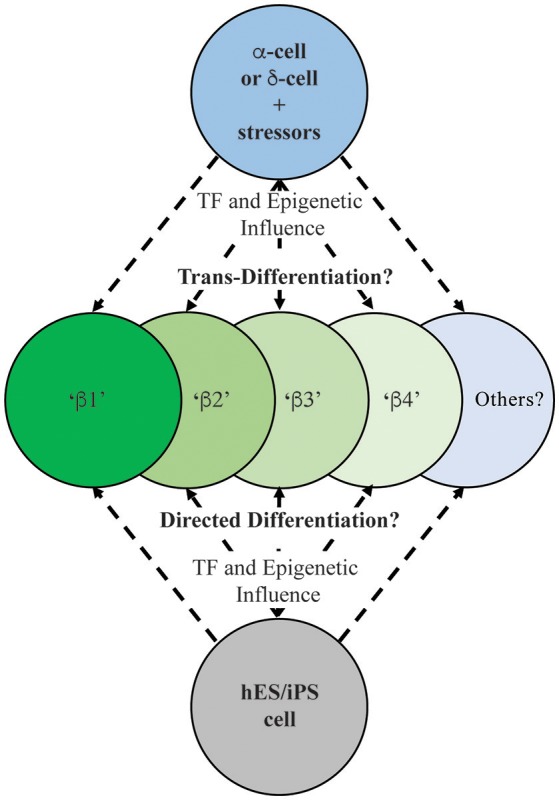
**Schematic illustrating the possible outcomes on human insulin-positive cell production during islet (e.g., α- or δ-cell; Thorel et al., 2010; Chera et al., 2014) and ES/iPS cell differentiation (Pagliuca et al., 2014; Rezania et al., 2014)**. Extensive characterization needs to be performed on these newly-generated insulin-producing cells to determine their functional and molecular characteristics in relation to existing human islet β-cell populations.

Underlying the notion of heterogeneity is whether human islet cells exist in a fixed, terminally differentiated state or if a level of cellular plasticity exists. Thus, evidence has been accumulating from studies in animal models that islet cells have the capacity to directly *trans*-differentiate to another islet cell fate and/or de-differentiate to a progenitor-like cell. The past decade has revealed in numerous rodent models that various stressors can impart pancreatic cell reprogramming, although it has been argued that β-cell replacement by replication is the predominant mechanism (Dor et al., 2004; Teta et al., 2007). Some physiological stressors, including pregnancy or obesity, have not provided clear evidence of islet cell reprogramming (reviewed in Cigliola et al., 2016). However, it appears that extreme physical injury, chemical stresses, or genetic perturbations are able to promote reprogramming (Figure 2). For example, as a model of pancreatitis, pancreatic duct ligation (PDL) causes regeneration of islets from Ngn3^+^ transcription factor islet endocrine progenitors adjacent to the ductal epithelium (Xu et al., 2008). This level of regeneration does not speak to the plasticity of β-cells *per se*, but highlights how the pancreas (or resident progenitors) can combat injury.

Significantly, direct α-to-β or β-to-α conversion has been observed through lineage tracing in several cell-type specific transcriptional regulator gene deficiency or over-expression models, including *Arx, Pdx1, Pax4*, and *Dnmt1* (Collombat et al., 2007, 2009; Dhawan et al., 2011; Yang et al., 2011; Gao et al., 2014). Cell ablation studies employing β-cell toxins including diphtheria toxin, streptozotocin, or alloxan, have also been useful in demonstrating how other islet endocrine cell types can adopt β-cell fates (Figure 2). For example, the Herrera group in Switzerland demonstrated that a nearly complete loss of islet β-cells imposed by directed diphtheria toxin destruction results in the reprogramming of adult islet α-cell or juvenile islet δ-cells to a β-cell fate (Thorel et al., 2010; Chera et al., 2014). Also, administration of the β-cell toxin alloxan in combination with PDL or the cerulein peptide (an inducer of pancreatitis) promoted *trans*-differentiation of α- to β-cells (Chung et al., 2010; Piran et al., 2014). Collectively, these results demonstrated that rodent islet cells have the capacity to *trans*-differentiate and replace β-cell mass. However, few of these studies have included comprehensive molecular and functional analysis to determine how closely reprogramming represents the *bona fide* islet β-cell populations.

It is likely that the mechanisms underlying how islet cell types adopt β-cell-like fates involve epigenetic influences at critical islet loci. For example, permissive histone and/or DNA modifications (e.g., methylation) may allow for expression β-cell-specific genes in α-cells, thus promoting cellular conversion. Bramswig et al. employed RNA- and ChIP-sequencing in sorted human α- and β-cells to reveal a remarkable level of α-cell plasticity, by examining the levels of the activating histone H3K4me3 and repressive H3K27me3 marks (Bramswig et al., 2013). Surprisingly, α-cells bear a large number of β-cell genes bivalently marked by H3K4me3 and H3K27me3, suggesting these genes are in a poised, inducible state. These included key β-cell functional genes, like *MAFA, PDX1, GLP1R*, and *PCSK1*. Furthermore, treatment of whole human islets with Adox, a histone methyltransferase inhibitor that prevents H3K27me3 formation, induced β-cell-specific *PDX1* expression in α-cells. Indeed, ultrastructural analysis revealed insulin and glucagon granule co-localization in Adox-treated cells. When jointly considered, these data suggest that α- (and likely δ-) cells exist in a malleable epigenomic state that enables conversion to β-like cells in many mouse models (Collombat et al., 2009; Thorel et al., 2010; Yang et al., 2011), and potentially in human islets (Bramswig et al., 2013) (Figure 2).

## De-differentiation and T2D

The apparent heterogeneity of the islet β-cell population and plasticity of other islet cell types led the field to question what happens to β-cell identity during the progression to T2D state. Under conditions of prolonged T2D, β-cells likely die, leading to reduced β-cell mass, but recent studies suggest that de-differentiation may also cause loss of insulin^+^ cell mass. In this regard, one of the most striking observations came from Talchai et al. (2012) examining how β-cell fate was affected in FoxO1-deficient mice under the metabolically challenging and stressful conditions associated with high fat diet and multiple pregnancies. Rather than islet β-cell dysfunction and death, they found that β-cells apparently de-differentiated, as defined by insulin degranulation, and most significantly, upregulation of various embryonic progenitor markers (e.g., Ngn3, L-Myc, Nanog, and Oct4; Talchai et al., 2012) (Figure 1). Work from the Melton group also revealed that loss of Urocortin 3 (Ucn3), a mature β-cell marker (Blum et al., 2012), is an early signal of de-differentiation in diabetic mouse models (e.g., *ob/ob, db/db, Akita*) (Blum et al., 2014). Furthermore, the progenitors arising from the de-differentiated β-cell pool in the Talchai et al. study (i.e., insulin^−^/Ngn3^+^/Oct4^+^/L-Myc^+^ cells) were followed by lineage tracing to reveal their conversion to non-β-cell fates, including α-, δ, and PP-cells. A similar population of de-differentiated β-cells were found in other T2D mouse models, including *db*/*db* and GIRKO (*Glut4*-*Cre* mediated *InsR* knockout; Lin et al., 2011) mice. In two other complementary studies, diabetic mice bearing activating K_ATP_ channel mutations lead to increased expression of *Ngn3* and production of insulin^−^ β-cells (Brereton et al., 2014; Wang et al., 2014). Strikingly, insulin treatment of these diabetic mice not only restored glucose homeostasis, but resulted in re-differentiation of the Ngn3^+^ cells into insulin^+^ β-cells. Collectively these studies not only suggest that a key outcome of T2D is β-cell de-differentiation, but that the process is reversible (Figure 1).

These reports revealed that de-differentiation and re-differentiation as mechanisms of islet β-cell failure and rescue, however the therapeutic importance would only be of significance if observed in human T2D β-cells. To this end, evidence for this possibility is unclear or even unsupportive, which may in part be due to the relatively recent nature of these observations in mice, but may also reflect differences between species (Kim et al., 2009; MacDonald et al., 2011; Dai et al., 2012). Nonetheless, some promising observations have been reported. White et al. employed immunofluorescence examination of three human T2D donor pancreata to reveal numerous insulin^+^ cells that co-localized with glucagon or the vimentin mesenchymal marker (White et al., 2013), suggesting altered β-cell identity. Accordingly, Spijker et al. found a significant increase in bihormonal insulin^+^/glucagon^+^ and NKX6.1^+^/amyloid^+^/glucagon^+^ cells upon analyzing mature β-cell markers (e.g., MAFA, FOXO1, NKX6.1) in human and non-human primate normal or T2D samples (Spijker et al., 2015). These “mixed phenotype” cells are consistent with the loss of β-cell and gain of α-cell characteristics in T2D islets. Similarly, the Accili group at Columbia University suggested that the presence of insulin^−^/synaptophysin^+^/ALDH1A3^+^ cells in T2D islets implies that de-differentiation is occurring under these conditions (Cinti et al., 2016), although ALDH1A3 could also be marking dysfunctional islet β-cells. Moreover, the stomach hormone gastrin, a typical embryonic pancreas marker, was found to be induced in mouse and human β-cells under hyperglycemic T2D conditions, a phenomenon that resolves upon glucose normalization (Dahan et al., 2016).

While the above results indicate that β-cells may dedifferentiate under T2D conditions, others have argued that this is minor, or even non-existent. Thus, Butler et al. recently posited that the hormone-negative chromogranin^+^ cells observed in T2D pancreata are not the dedifferentiated β-cells found in mouse models, but rather immature β-cells arising during regeneration (Butler et al., 2016). Moreover, while Guo et al. (2013) found a change in β-cell identity was supported by the loss in many cell-enriched transcription factors in human T2D islets, there was no evidence for the increased progenitor mRNAs expression found in rodent studies, including *NGN3, NANOG*, and *MYCL1*. The human islet single-cell transcriptome analyses have also failed to reveal compelling evidence for de-differentiation (Segerstolpe et al., 2016; Xin et al., 2016). It is very likely that the extensive efforts underway to characterize normal and T2D human islet populations will soon resolve how islet β-cell function is compromised in the T2D patients, and hopefully lead to the development of therapeutics to either prevent or correct the deficiencies.

## Conclusions and perspectives

The possibility that human T2D β-cells undergo de-differentiation, *trans*-differentiation, or (at least) loss of identity raises several questions about future diabetes therapeutic strategies. Can we exploit the signals that trigger β-cell de-differentiation or loss of identity during these processes? Specifically, can drugs or small molecules be designed to attenuate the process, or that efficiently and specifically regenerate functional β-cells? These studies could be similar to small molecule screens that have revealed that harmine analogs positively impact human β-cell proliferation (Wang et al., 2015). Blum et al. also employed an *in vivo Ucn3-GFP* reporter and lineage tracing to track the de-differentiation state of mouse β-cells in the presence of various small molecules (Blum et al., 2014). A TGFβ pathway inhibitor, Alk5 inhibitor II, potently restored the GFP signal in islets cultured under de-differentiation conditions. Further, the compound increased mRNA levels of key β-cell maturity markers including *Ucn3, MafA, Nkx6*.1, and *Pdx1*. However, much more work needs to be done to definitively demonstrate whether these mechanisms are at play in human β-cells. If so, the field should focus on developing pharmacological reagents that can maintain the mature, differentiated β-cell, or induce re-differentiation in T2D patients. Further, there is a need for more extensive studies revealing the unique properties regulating the activity of the distinct β-cell populations produced under normal and diabetic circumstances. Given the notable morphological and functional differences between rodent and human islets (Kim et al., 2009; MacDonald et al., 2011; Dai et al., 2012), improved modeling of the human disease process will also move the field forward. A recent report from the Powers group at Vanderbilt University took strides toward addressing this by comparing transplanted mouse and human islets under gluco- or lipo-toxic conditions, revealing significant differences in responses between species (Dai et al., 2016). The ability to lineage trace human islet cells would also be a major innovation allowing for a better understanding of the fate of β-cells when exposed to diabetic stress conditions *in vivo*. Importantly, recent studies have brought to the forefront that not all insulin^+^ cells are equal, and increasing the mass and/or activity of truly functional (i.e., glucose sensing and insulin secreting) β-cells will push the field closer to a diabetes cure. Moreover, assessing levels of such heterogeneity markers (e.g., ST8SIA1, CD9, Fltp, ALDH1A3) during human embryonic stem cell (or induced pluripotent stem cell) directed β-cell differentiation protocols (Pagliuca et al., 2014; Rezania et al., 2014) (Figure 2), including cells currently in clinical trials (Viacyte, clinicaltrials.gov identifier NCT02239354), will allow for a better evaluation of the integrity, functionality and reliability of these potential therapeutic reagents.

## Author contributions

CH and RS each conceived, designed, wrote, and edited this manuscript.

### Conflict of interest statement

The authors declare that the research was conducted in the absence of any commercial or financial relationships that could be construed as a potential conflict of interest.
